# Memory function and early exit from paid employment through different pathways among ageing European workers

**DOI:** 10.5271/sjweh.4211

**Published:** 2025-03-01

**Authors:** Robert Ciliacus, Roos W Hijdra, Suzan JW Robroek, Anja K Leist, Alex Burdorf, Merel Schuring

**Affiliations:** 1 Erasmus University Medical Center Rotterdam, Department of Public Health, Rotterdam, The Netherlands.; 2 University of Luxembourg, Department of Social Sciences, Esch-sur-Alzette, Luxembourg.

**Keywords:** cognition, disability pension, early retirement, effort–reward imbalance, ERI, psychosocial work characteristics, unemployment, workforce participation

## Abstract

**Objectives:**

Understanding memory function’s role in early workforce exit is key in supporting sustainable employment among ageing workers. This study examined the impact of memory function on early exit from paid employment, analyzed changes in memory function before, during and after such transitions, and assessed memory function trajectories in relation to the presence or absence of effort-reward imbalance at work.

**Methods:**

This study included 16 339 respondents from the Survey of Health, Ageing, and Retirement in Europe (SHARE) between age 50 and the country-specific retirement age. The effects of objective and subjective memory functioning on early exit were assessed using Cox proportional hazards with Fine-Gray sub distribution models. Changes in memory function before and after a transition to non-employment were assessed using generalized linear mixed-effects models. These changes were described and compared based on exposure to job effort–reward imbalance.

**Results:**

Workers with poor subjective memory were 2.3 times more likely to exit employment prematurely due to disability [sub-distribution hazard ratio (SHR) 2.30, 95% confidence interval (CI) 1.77–3.00] and 1.3 times more likely to exit through unemployment (SHR 1.29, 95% CI 1.06–1.55). Workers with low objective memory were 1.6 times more likely to exit through unemployment (SHR 1.56, 95% CI 1.30–1.87). Subjective memory generally declined prior to, and during early exit from paid employment. While subjective memory generally improved post-exit, objective memory function declined after exiting. An accelerated decline in objective memory functioning was noted among early retirees who had been exposed to effort–reward imbalance at work (β -0.45, standard error 0.16).

**Conclusion:**

Workers with poor memory function are at higher risk of early involuntary exit from paid employment. Promoting memory function and balancing job efforts and rewards may help mitigate the risk of a premature exit.

Europe is facing challenges inherent to an ageing population and the subsequent shrinkage of its workforce. In response, many European countries are proactively raising their statutory retirement age and implementing measures to extend working lives (1). The success of such measures depends on a comprehensive understanding of the complexities of ageing in the workforce, particularly regarding specific age-related health issues that may impede ageing workers’ ability to maintain paid employment. As the workforce ages, there is a corresponding increase in the prevalence of age-related health issues, including memory impairment (2). With growing expectations for individuals to work until later stages of life, it is imperative to examine the role of memory function in the ability of ageing workers to maintain paid employment.

While certain cognitive aspects, such as verbal ability and cumulative knowledge, tend to improve with age, declines are generally observed in domains requiring intensive processing, including memory function (3). As a result of normal cognitive ageing, modest declines in memory are generally observed up to the age of 65, after which the decline accelerates (4). Older adults with better memory function generally tend to report better self-perceived health outcomes (5). In contrast, memory impairment can disrupt individuals’ sense of well-being, reduce functional and occupational capacity, and contribute to other adverse health outcomes (6–9). Consequently, older individuals with memory impairment may become vulnerable to exiting the workforce prematurely due to poor health (10, 11). A departure from the workforce at a later age is often final, as re-entering the workforce has proven rare, particularly for those who exited due to disability or opted for early retirement (12).

This raises concerns, as exiting paid employment may additionally adversely affect subsequent memory function (13–15), particularly among individuals who exit prematurely (16, 17). Conversely, prolonged participation in paid employment appears to serve a protective role in preserving memory function (18, 19). Promoting continued participation in paid employment at an older age could therefore serve as a reasonable strategy to protect the memory function of ageing workers, while simultaneously ensuring sustainability of the workforce. However, poor memory function may result in a misalignment between an individual’s capabilities and job requirements, potentially prompting an early exit from paid employment. Such misalignment may be further exacerbated by unfavorable working conditions.

Enhancing sustainable employment among ageing workers facing health issues, such as memory impairment, involves optimization of the psychosocial work environment (20). While certain psychosocial work conditions, such as high job control and complexity, are known to protect against cognitive decline, other conditions may, in turn, increase the risk of cognitive decline in the long term (21). Specifically, working conditions that evoke chronic stress are likely to affect different aspects of cognitive processing, including memory function (21, 22). Stress may arise from work characterized by high effort, combined with low rewards. The effort–reward imbalance (ERI) model provides a theoretical and analytical framework for estimating such experienced work-related stress (23, 24). Job ERI may shape ageing workers’ memory trajectories both before and after exit from paid employment. Still, the extent to which job ERI influences memory function over time remains uncertain. A better understanding of these dynamics is essential in order to support the development of effective interventions and policies supporting the continued work participation of ageing workers.

The influence of memory function on ageing workers’ ability to maintain employment remains poorly understood. Research shows that subjective memory complaints do not significantly correlate with objective memory measures (25). Therefore, in order to gain a comprehensive insight into the interplay between memory function and early exit from paid employment, it is key to consider both objective and subjective memory function separately. While objective assessments of memory function help evaluate actual cognitive performance, they may not fully capture the everyday challenges individuals face due to memory limitations. In contrast, self-reported memory offers valuable insights into how individuals perceive their cognitive abilities, though these perceptions are likely influenced by their day-to-day memory demands.

This study therefore aims to provide insight into (i) the influence of objective and subjective memory function on early exit from paid employment through different pathways among ageing workers in Europe, (ii) changes in objective and subjective memory function before, during, and after early exit from paid employment. It also aims to (iii) describe and compare objective and subjective memory function trajectories before, during and after early exit from paid employment in the presence or absence of exposure to job ERI pre-exit.

## Methods

### Study design and population

This longitudinal study investigated the interplay between memory function and early workforce departure among ageing workers in European countries. The study utilized data from the Survey of Health, Ageing, and Retirement in Europe (SHARE), a research infrastructure designed to study the effects of health, social, economic and environmental policies over the life course of European citizens (26). SHARE targets individuals aged ≥50 years and their respective partners regardless of age. Its coverage has expanded over time, currently including data from 27 European countries, plus Israel. Data is collected biannually through computer-assisted personal interviews. Sampling designs varied from random sampling utilizing national population registries to multi-stage sampling employing regional or local population registers (27). For detailed information concerning data collection, data comparability, and response rates, please refer to the official SHARE documentation available at share-eric.eu/data/data-documentation.

The Ethics Committee of the University of Mannheim (waves 1 – 4) and the Ethics Council of the Max Planck Society (waves 4 – 7) reviewed and approved SHARE. The Medical Ethical Committee of Erasmus MC, Rotterdam approved the current study (MEC-2023-0568).

The current study utilized data from SHARE waves 4 (2011/12), 5 (2013), 6 (2015), 7 (2017), and 8 (2019/2020). Waves 1–3 were omitted as subjective memory was assessed only from wave 4 onwards. In the supplementary material (www.sjweh.fi/article/4211), figure S1 illustrates the participant flow, starting with 118 851 individuals who reported their current employment status. Individuals were excluded if at baseline they: (i) were not employed (N=83 469), (ii) were not aged between 50 and the country-specific statutory retirement age (N=5131), (iii) did not take part in the cognitive function test module (N=5867), or (iv) did not participate in two consecutive waves (N=8045). As a result, the final sample for the survival analyses consisted of 16 339 workers, aged between 50 and the statutory retirement age, who provided data on memory function for at least two consecutive waves. Subsequently, respondents who exited paid employment early due to early retirement, disability or unemployment (N=3816) were included in the generalized linear mixed-effects model for further analysis (subpopulation I). Additionally, 2406 respondents who exited paid employment through early retirement, disability, or unemployment and provided information on their perceived psychosocial work environment were included in subpopulation II.

### Measures

*Subjective and objective memory.* Two measures of memory function were used: subjective and objective. Subjective memory was assessed using a single-item question asking “How would you rate your memory at the present time?” using a 5-point Likert scale, ranging from “excellent” to “poor”. For the survival and mixed-effects models, subjective memory was operationalized as a categorical variable and continuous variable respectively. Subjective memory was categorized into three categories: (i) very good or excellent, (ii) good, and (iii) fair or poor. The continuous variable was created by rescaling the 5-point Likert scale to a range of 1–100.

Objective memory was assessed using two single items derived from the larger Harmonized Cognitive Assessment Protocol (HCAP), a validated tool for cross-national comparison of later-life cognitive function (28, 29). The HCAP includes an evaluation of immediate recall function (promptly recalling words from a ten-word list) and delayed recall function (recalling the ten-word list after a short delay). Although these episodic memory tests have not been validated as a standalone measure, similar assessments have been extensively used in previous studies to evaluate both cognitive and memory function (14, 16, 17, 19, 30, 31).

A composite measure for objective memory was derived by averaging the total words recalled in both tests, resulting in a score within a 0–10 scale. Objective memory was categorized into three levels – low, intermediate, or high – using tertiles to establish cut-offs. The continuous variable was created by rescaling into a 0–100 scale to facilitate comparability between subjective and objective memory assessments.

### Early exit from paid employment

Early exit from paid employment was defined as departing paid employment before reaching the country-specific statutory retirement age. Employment status was assessed at each observation using a single item question asking “In general, which of the following best describes your current employment situation?” The answer categories were: retired, employed or self-employed, unemployed and seeking work, permanently sick or disabled, home-maker, or other. An early exit from paid employment was identified as the transition from ‘employed or self-employed’ in wave x-1 to ‘retired’ (early retirement), ‘permanently sick or disabled’ (disability), ‘unemployed’ (unemployment), or ‘homemaker’ (economic inactivity) in wave x before reaching the statutory retirement age. While early retirement was considered to be a voluntary exit pathway from paid employment, unemployment and disability were considered involuntary exit pathways. Country- and sex-specific statutory retirement ages during the selected waves were derived from the OECD’s “Pensions at a Glance” reports (32–36). An overview of the country-, time- and sex-specific statutory retirement ages for each country is provided in supplementary table S2.

### Effort–reward imbalance at work

ERI was measured at baseline, utilizing seven items from the ERI questionnaire (24). Job effort was operationalized through two items assessing perceived physical demand and time pressure. Job reward was assessed using five items encompassing perceived social support, recognition, earnings, advancement prospects, and job security (37). Participants were asked to indicate their level of agreement with statements (eg, “My job is physically demanding”) on a 4-point Likert scale, ranging from 1 (strongly disagree) to 4 (strongly agree). Effort and reward sum scores were determined by summing the corresponding items. A lower score is more favorable for job effort, whereas a higher score is more favorable for job reward. The ERI ratio was computed using the formula effort/reward×c, where “c” is the correction factor (c=0.4) accommodating the different number of items used to calculate effort and rewards. Subsequently, the ratio was dichotomized, with values >1.0 signifying an imbalance between effort and reward (38).

### Sociodemographic characteristics

Sociodemographic characteristics included sex, age, and education level. Age was categorized into three categories: 50–54, 55–59, and ≥60 years. The International Standard Classification of Education (ISCED-97) was utilized to classify education levels as low (pre-primary, primary, and lower-secondary), intermediate (upper-secondary), or high (post-secondary and tertiary).

### Statistical analysis

Cox proportional hazards analyses were conducted to analyze the influence of individual characteristics, memory function and ERI of workers on early exit from employment. Participants were censored from the analyses upon reaching the statutory retirement age, when lost to follow-up or at the end of the follow-up period (wave 8).

Subsequently, the Fine-Gray model was employed to assess the impact of memory function early exit from the workforce through distinct (competing) pathways (39). Subjective and objective memory function were included in the model separately. Workers who exited (self-) employment through a specific pathway (e.g., unemployment) were compared with individuals who either maintained employment or exited through different pathways (e.g., disability and early retirement). Therefore, the calculated sub-distribution hazard ratio (SHR) for exiting through each specific pathway can be interpreted as the absolute risk, taking the possibility that competing events may precede the occurrence of the event of interest into account (40). The analyses were adjusted for age, sex and education level.

Furthermore, trajectories of both subjective and objective memory were examined among individuals who experienced an early departure from the workforce using generalized linear mixed-effects models. Changes in memory function before, during and after a transition from employment into early retirement, unemployment or disability were analyzed. Utilizing the regression model outlined below, the changes in memory over time were estimated before and after individuals exited the workforce prematurely:

Y_it_ = β_0_ + β_1_(time_before) + β_2_(before_after) + β_3_(time_after) + β_4_ (age) + β_5_ (sex) + β_6_ (edu) +e_t_.

Y_it_ represents the dependent variable, which can be either subjective or objective memory of individual ‘i’ at time ‘t’. β_0_ represents the intercept, estimating the expected value of both memory outcomes when all independent variables in the model are zero. β_1_ estimates the annual change in memory before an early exit from the workforce has occurred. β_2_ represents the step-change in memory during the (two-year) transition from employment (before_after = 0) to non-employment (before_after = 1). β_3_ estimates the annual change in memory outcomes after an early exit from the workforce has occurred. The analyses were adjusted for age during the transition, sex and education level. A random intercept was incorporated in the model to account for the variability in the outcome attributable to differences between individuals. Additionally, changes in memory outcomes before, during and after early exit from paid employment were analyzed among individuals with or without job ERI at work. Interaction terms were included to compare memory trajectories between the groups, assessing whether annual changes in memory function (before, during and after the transition) differed significantly between individuals with or without previous exposure to ERI at work.

All analyses were performed in Stata 18 (Stata Corp, College Station, TX, USA), using the “stcox” command for the survival analysis, the “stcrreg” command for the competing risk analyses, and the “xtmixed” command for the generalized linear mixed-effects models.

## Results

The survival analyses included 16 339 participants, with an average follow-up time of 4.2 [standard deviation (SD) 2.3] years, and a maximum follow-up time of 9.3 years. At baseline, one in seven participants (13.7%) rated their memory as fair or poor, whereas one third of the participants (31.5%) obtained a low objective memory score. Subjective memory showed a weak positive correlation [r=0.16, 95% confidence interval (CI) 0.14–0.17] with objective memory at baseline. Among participants who provided information on work characteristics, 36.1% reported ERI at work. A quarter (N=4158; 25.4%) of all participants exited the workforce before reaching the statutory retirement age. The most common exit pathway out of paid employment was early retirement (59.2%), followed by unemployment (21.3%), disability (11.2%) and economic inactivity (8.2%). Due to its small sample size, results for the group categorized as economically inactive are not presented.

Table 1 shows that older individuals and individuals with lower levels of education were more likely to experience early exit from paid employment. Individuals with poor (HR 1.37, 95% CI 1.25–1.50) and good (HR 1.23, 95% CI 1.15–1.31) subjective memory were at increased risk of exiting the workforce prematurely compared to individuals who reported very good or excellent subjective memory. Similarly, individuals with low (HR 1.56, 95% CI 1.44–1.68) or intermediate (HR 1.20, 95% CI 1.11–1.30) objective memory were more likely to exit paid employment early compared to those achieving high objective memory scores. Furthermore, ERI at work was associated with an increased likelihood of early exit from paid employment (HR 1.29, 95% CI 1.19–1.39).

**Table 1:** Baseline individual and work-related characteristics and their impact on early exit from paid employment among 16 339 ageing European workers. **Bold: statistically significant at the 0.05 level.**

		Total (N=16 339)			Early exit from paid work All pathways ^a^
		N	%		HR (95% CI)
Age categories					
	50–54	7210	44.1		1
	55–59	6374	39.0		**2.02 (1.89–2.16)**
	≥60	2755	16.9		**2.04 (1.86–2.24)**
Sex					
	Female	8377	51.3		1
	Male	7962	48.7		1.01 (0.95–1.08)
Education					
	Post-secondary and tertiary	6435	39.4		1
	Upper secondary	6353	38.9		**1.49 (1.39–1.61)**
	Up to lower secondary	3365	20.6		**2.20 (2.04–2.39)**
	Missing	186	1.1		
Subjective memory					
	Very good or excellent	6936	42.5		1
	Good	7169	43.9		**1.23 (1.15–1.31)**
	Fair or poor	2234	13.7		**1.37 (1.25–1.50)**
Objective memory					
	High (>60)	4924	30.1		1
	Intermediate	6272	38.4		**1.20 (1.11–1.30)**
	Low (<50)	5143	31.5		**1.56 (1.44–1.68)**
Effort–reward imbalance model					
	No effort–reward imbalance	5572	34.1		1
	Effort–reward imbalance	3146	19.3		**1.29 (1.19–1.39)**
	Missing	7621	46.6		

^a^ Univariate Cox proportional hazard analyses.

Table 2 demonstrates that poor memory function increased the likelihood of exiting the workforce early through disability and unemployment. Individuals who reported poor or fair subjective memory were 2.3 times more likely to exit due to disability (SHR 2.30, 95% CI 1.77–3.00) and 1.3 times more likely to become unemployed (SHR 1.29, 95% CI 1.06–1.55) compared to individuals who reported very good or excellent subjective memory. Likewise, workers with a low objective memory score were 1.6 times more likely to exit paid employment early due to unemployment (SHR 1.56, 95% CI 1.30–1.87). Neither lower subjective nor objective memory function was associated with a significantly increased likelihood of early retirement.

**Table 2:** The impact of subjective and objective memory function on early exit from paid employment through different pathways among 16 339 ageing European workers. **Bold: statistically significant at the 0.05 level.**

		Early exit from paid employment ^a^				
		Early retirement (N=2465)		Disability (N=466)		Unemployment (N=885)
		SHR (95% CI)		SHR (95% CI)		SHR (95% CI)
Subjective memory						
	Very good or excellent	1		1		1
	Good	1.02 (0.94–1.12)		**1.78 (1.43–2.22)**		1.02 (0.88–1.18)
	Poor or fair	0.95 (0.84–1.08)		**2.30 (1.77–3.00)**		**1.29 (1.06–1.55)**
Objective memory						
	High	1		1		1
	Intermediate	1.03 (0.93–1.11)		1.06 (0.84–1.34)		**1.21 (1.02–1.45)**
	Low	1.10 (0.99–1.22)		1.17 (0.91–1.50)		**1.56 (1.30–1.87)**

^a^ Fine-Gray analyses (adjusted for age, sex and education level).

Approximately a quarter (N=3816; 23.4%) of all participants exited the workforce prematurely due to early retirement, disability or unemployment and were included in the generalized linear mixed-effects analyses (subpopulation 1). The mean follow-up time pre-transition out of paid employment was 2.7 years (maximum 8.2 years), and the mean follow-up post-transition was 2.9 years (maximum 8.1 years). Mean subjective memory at baseline for all workers who exited paid employment prematurely was 57.24 (SD 22.21), while mean objective memory at baseline was 52.7 (SD 15.67) (supplementary table S1).

**Figure 1:**
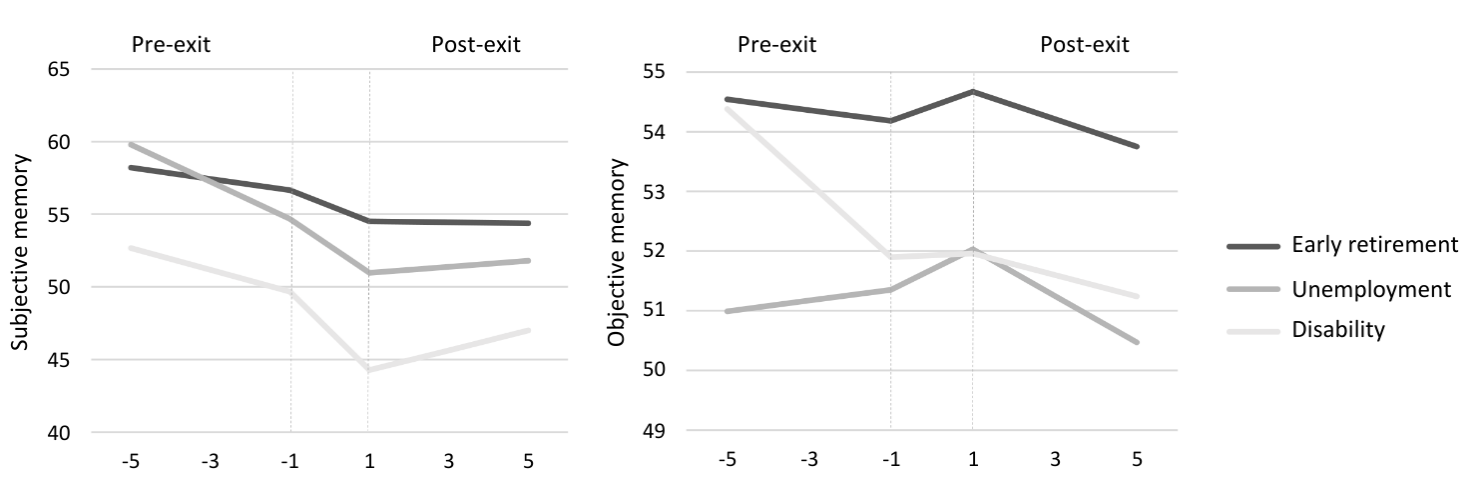
Changes in memory function before and after early exit from paid employment (unadjusted).

Table 3 and figure 1 show the changes before, during and after early exit from paid employment through distinct pathways. A decline in subjective memory prior to exit from paid employment was noted irrespective of the pathway of exit. The steepest annual decline in subjective memory was found prior to unemployment (β -1.28, SE 0.38), followed by disability (β -0.70, SE 0.47) and early retirement (β -0.38, SE 0.17). During the (two-year) period of the transition, a negative (two-year) step-change in subjective memory was evident among all workers who exited the workforce prematurely. The step-change was highest among workers who exited through involuntary pathways: disability (β -5.45, SE 2.03) and unemployment (β -2.77, SE 1.49). After exiting employment, subjective memory stabilized among individuals who transitioned to early retirement (β -0.02, SE 0.17), and improved among individuals who transitioned to unemployment (β 0.31, SE 0.33) and disability (β 0.69, SE 0.41).

**Table 3:** Changes in memory before, during and after an early exit from paid employment among 3996 ageing European workers. **Bold: statistically significant at the 0.05 level.**

		Early exit from paid employment ^a^				
		Early retirement N=2465		Disability N=466		Unemployment N=885
		β (SE)		β (SE)		β (SE)
Subjective memory (1–100)						
	Annual change before transition	**-0.38 (0.17)**		-0.70 (0.47)		**-1.28 (0.38)**
	Step-change during transition	**-1.77 (0.88)**		**-5.45 (2.03)**		**-2.77 (1.49)**
	Annual change after transition	-0.02 (0.17)		0.69 (0.41) ^b^		0.31 (0.33) ^b^
Objective memory (0–100)						
	Annual change before transition	-0.08 (0.10)		-0.50 (0.29)		0.21 (0.21)
	Step-change during transition	**0.80 (0.39)**		0.66 (1.00)		0.83 (0.07)
	Annual change after transition	**-0.24 (0.09)**		-0.17 (0.23)		**-0.35 (0.17)** ^b^

^a^ Generalized linear mixed-effect models (adjusted for age, sex and education level). ^b^ Annual rate of change before and after exiting is significantly different at the 0.05 level.

In contrast, no significant changes in objective memory functioning were found prior to an early exit from paid employment. Irrespective of the pathway of exit, individuals experienced an increase in objective memory functioning in the two-year period during which the employment transition took place. After transitioning to non-employment, individuals experienced a decline in objective memory, regardless of the pathway of exit. This decline was most pronounced among individuals who exited the workforce due to unemployment (β -0.35, SE 0.17) and early retirement (β -0.24, SE 0.09) (table 3 and figure 1). Following an early exit due to unemployment, the annual change in objective memory function significantly worsened compared to the annual change prior to exiting.

**Figure 2:**
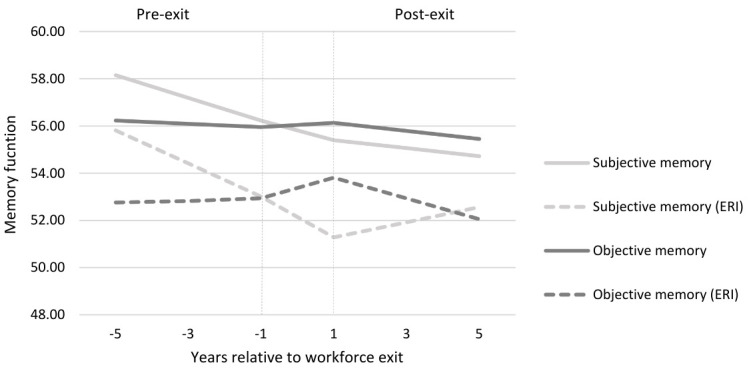
Memory function trajectories of early retirees: the impact of job effort–reward imbalance (ERI) (unadjusted).

Of the participants who exited paid employment early due to early retirement, disability or unemployment, 2406 provided information regarding their psychosocial work environment (subpopulation 2). The prevalence of perceived ERI at work was highest among individuals who exited paid employment through involuntary pathways. Specifically, 50.5% of workers who exited paid employment due to disability and 48.7% of workers who exited due to unemployment, reported ERI at work (supplementary tables S3 and S4). In contrast, among workers who opted for early retirement, 37.6% encountered job ERI at work (table 4). Workers with ERI at work reported lower subjective memory and lower objective memory at the time of transition, regardless of their exit route, compared to those who did not experience ERI (figure 2, supplementary figures S2 and S3).

**Table 4:** Changes in memory before and after early retirement: the role of job effort–reward imbalance among 1573 early retirees. **Bold: statistically significant at the 0.05 level.** [SE=standard error.]

		N (%)	Memory function ^a^		
			Subjective (1–100)		Objective (0–100)
			β (SE)		β (SE)
Effort–reward imbalance		592 (37.6)			
	Annual change before transition		**-0.73 (0.33)**		0.07 (0.19)
	Step-change during transition		-1.18 (1.67)		1.28 (0.79)
	Annual change after transition		0.32 (0.30)		**-0.45 (0.16)**
No effort–reward imbalance		981 (62.4)			
	Annual change before transition		**-0.48 (0.23)**		-0.04 (0.13)
	Step-change during transition		-0.16 (0.22)		0.40 (0.59)
	Annual change after transition		-0.20 (1.31)		-0.20 (0.12)

a Generalized linear mixed-effect models (adjusted for age, sex and education level).

Table 4 and figure 2 further illustrate that workers experiencing ERI reported a greater decline in subjective memory before early retirement (β -0.73, SE 0.33) compared to those who did not (β -0.48, SE 0.23), although the difference was not statistically significant. Changes in subjective memory function following an early transition out of paid employment, generally did not significantly differ between individuals with and without previous exposure to ERI at work. However, early retirees who were previously exposed to ERI at work experienced a small annual improvement in subjective memory after transitioning to early retirement (β 0.32, SE 0.30), while subjective memory functioning among unexposed workers continued to decline (β -0.20, SE 1.31). Similar trends were found among workers with ERI who exited through involuntary pathways (supplementary tables S3 and S4). Objective memory functioning declined after a transition to early retirement irrespective of the former psychosocial work environment. An accelerated decline was noted among workers who had been exposed ERI at work (β -0.45, SE 0.16) compared to those without ERI (β -0.20, SE 0.12), although the difference was not statistically significant.

## Discussion

Both poor subjective and objective memory function increased the likelihood of early exit from paid employment through disability or unemployment among ageing European workers. Workers with poor subjective memory were 2.3 times more likely to exit prematurely due to disability and 1.3 times more likely to exit through unemployment. Workers with low objective memory were 1.6 times more likely to exit through unemployment. Subjective memory generally declined prior to, and during, an early exit from paid employment. After exit from paid employment due to disability or unemployment, a positive trend change in subjective memory was noted. Conversely, objective memory declined in the years after the transition into early retirement and unemployment. Early retirees who had been exposed to ERI showed a significant decline in objective memory after exiting paid employment, which was not observed among those without such imbalance. However, changes over time did not differ statistically significantly between individuals with and without ERI.

Poor subjective memory notably elevated the absolute risk of exiting paid employment early through unemployment and disability, indicating that individuals with low subjective memory are more vulnerable to an involuntary exit from employment. This aligns with previous research indicating that poor self-reported health is a risk factor for exiting paid employment, particularly through involuntary pathways (41). Poor objective memory was primarily associated with an increased risk of exiting early through unemployment. While poor memory may thus contribute to involuntary labor force exits, some evidence suggests that individuals with memory impairment can remain employed in a supportive work environment (42). Our findings indicate that the likelihood of exit from paid employment through early retirement is not driven by memory functioning. These findings support the notion that factors beyond impaired health, such as financial considerations and social factors, may play a more prominent role in voluntary retirement decisions (43).

While subjective memory declined prior to the transition, it stabilized or improved after transitioning to non-employment. In contrast, a decline in objective memory was noted after a transition to non-employment regardless of the pathway of exit. In congruence with previous research, accelerated objective memory decline was found within individuals after exiting paid employment (voluntarily) through early retirement (16). However, during the transition period, a short-lived increase in objective memory function was observed. These results concur with previous findings, suggesting that the effect of retirement on episodic memory is likely to manifest with delay (30). Indeed, previous research has shown that there seems to be a negative association between time spent in retirement and late-life memory function (19, 30, 31).

The deterioration observed in objective memory function, after transitioning from paid employment to early retirement or unemployment, supports the notion that retirement plays an important role in accounting for memory decline at older age. According to the use-it-or-lose-it hypothesis, intellectually engaging activities can stave off cognitive ageing, while a decrease in such activity patterns, for example due to non-employment, may result in the atrophy of cognitive skills such as memory function (17). However, our findings reveal a discrepancy between perceived and objective memory function following early exit from paid employment. As individuals who are no longer regularly engaged in cognitively demanding tasks may be less likely to notice subtle changes in memory function, subjective memory function may be overestimated. Moreover, considering that subjective memory function may be influenced by our emotional and motivational states (44), alleviating job-related stressors may positively impact psychological well-being, consequently improving individuals’ perception of their current memory function.

Individuals exiting jobs characterized by ERI generally left the workforce with lower subjective and objective memory function. Moreover, early retirees who were previously exposed to ERI experienced a significant decline in objective memory function after exiting paid employment, which was not found among those without imbalance. These results highlight the importance of work-related stress in cognitive ageing and corroborate previous findings suggesting that unfavorable psychosocial job characteristics may accelerate memory decline among ageing workers (45, 46). However, the annual rates of change in memory outcomes before, during and after early exit did not differ statistically significantly between individuals with and without ERI. Therefore, further research is needed to analyze the role of working conditions on changes in memory functions around the transition from paid employment. Our study suggests, with some caution, that ERI at work may not merely reduce the likelihood of a prolonged working life as demonstrated in the survival analysis, but it may furthermore affect memory function long-term, reaching far into the life post-retirement. Efforts to improve the balance between job efforts and rewards, and therefore reduce work-related stress, could reduce the likelihood of exiting the workforce prematurely while fostering larger memory reserve upon exit of the workforce.

Strengths of this study include its longitudinal design, which allows for the monitoring of memory function over time. Additionally, the cross-national scope and large size of the selected dataset, ensure generalizability of the results. Moreover, this study distinguishes between different exit routes out of paid employment, treating the distinct exit pathways as competing events. In doing so, it assesses the absolute risk for early workforce departures associated with poor subjective memory and low objective memory functioning.

However, some limitations need to be addressed. Firstly, all analyses were restricted to the first occasion in which a transition out of paid employment occurred. Re-entering paid employment was not taken into account, as most ageing workers who exit work remain out of the workforce, particularly after a transition to early retirement or disability (12). To disambiguate trajectories of individuals remaining non-employed from those who returned to work when estimating memory trajectories, observations were only considered valid as long as the individual remained non-employed. Previous SHARE studies have however reported that 25 to 30% of ageing workers who transitioned to unemployment do return to paid employment (20, 40). This indicates that a transition to unemployment is only temporary for some, and does not necessarily indicate a definite exit from the labor force.

Moreover, in this study, both employed and self-employed individuals are included in the study population. Early exit from employment may differ between these two groups due to variations in regulations. While employed individuals may benefit from (early) retirement schemes, self-employed individuals lack access to employer-sponsored retirement plans and are not always required to participate in public pension schemes. As a result, early retirement is often more financially demanding for the self-employed compared to employees. Further research is recommended to analyze differences between employed and self-employed individuals in the impact of memory function on early exit from paid employment as well as changes in memory function before, during and after such transitions in both groups.

In addition, ERI at work was measured through self-report. Self-reported measures (of psychosocial work factors) are inherently influenced by individuals’ subjective experiences. As such, the relationship between job-ERI and memory function is likely bidirectional. As memory deteriorates, individuals may feel like they are exerting more effort to perform their usual tasks. Therefore, they may feel that their job has become more demanding, even if the job requirements have not been altered. Therefore, the possibility of reversed causality needs to be acknowledged: memory function decline prior to the baseline assessment could have influenced participants’ perceptions of job effort and reward.

### Concluding remarks

In conclusion, workers with poor memory function are at increased risk of exiting paid employment before reaching the national statutory retirement age via involuntary exit pathways. Moreover, workers exposed to job ERI at work generally exit the workforce with lower memory reserve and tend to experience greater objective memory function decline after opting for early retirement. Efforts aimed at improving job efforts and rewards may reduce the likelihood of premature workforce exits, while fostering a larger memory reserve upon retirement. A work environment that promotes and maintains memory function at older age could potentially decrease the amount of workers exiting paid employment early through involuntary pathways, while ensuring sustainability of the workforce.

## Supplementary material

Supplementary material
