# Supplementary material for: Memory function and early exit from paid employment through different pathways among ageing European workers
Source: Scand J Work Environ Health. 2025 Feb 28;51(2):89–99. doi: 10.5271/sjweh.4211 (PMC11893212; doi:10.5271/sjweh.4211)
Supplement: Supplementary material [file SJWEH-51-89-S001.pdf]

Memory function and early exit from paid employment through different pathways among ageing European workers[1]

by Robert Ciliacus, MSc,[2] Roos W Hijdra, MSc, Suzan JW Robroek, PhD, Anja K Leist, PhD, Alex Burdorf, PhD, Merel Schuring, PhD

1. Supplementary tables & figures
2. Correspondence to: M Schuring, Erasmus University Medical Center Rotterdam, Department of Public Health, Rotterdam, the Netherlands. [E-mail: M.Schuring@erasmusmc.nl]

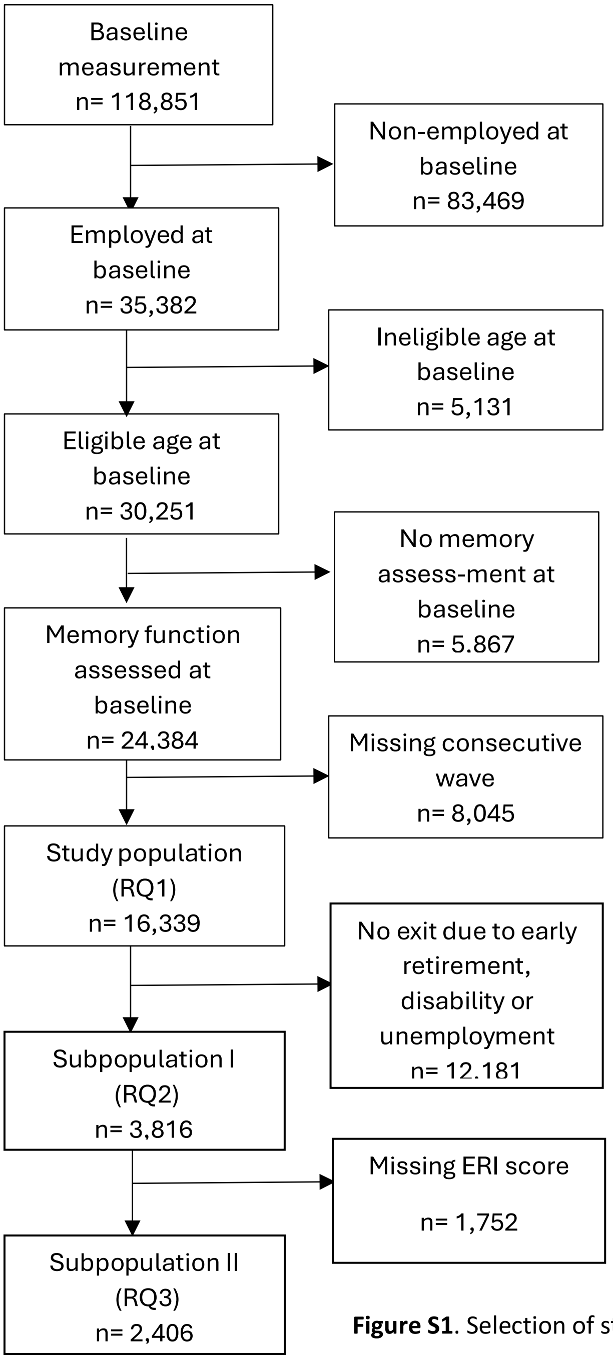

Figure S1. Selection of study population

Table S1. Average memory function at baseline among individuals who exit paid employment early.

	Early exit from paid employment			
	Early retirement	Disability	Unemployment	All
	Mean (SD)	Mean (SD)	Mean (SD)	Mean (SD)
**Subjective memory (1 – 100)**				
Baseline average	58.33 (21.98)	51.85 (21.05)	57.28 (23.38)	57.24 (22.21)
**Objective memory (0 – 100)**				
Baseline avarage	53.45 (15.37)	52.30 (16.34)	50.73 (15.77)	52.69 (15.67)

Table S2. Biannual country-, year- and sex-specific statutory retirement ages as defined by OECD's Pension At A Glance reports.

Country	SHARE ID	n	Statutory retirement age											
			2010		2012		2014		2016		2018		2020	
			M	F	M	F	M	F	M	F	M	F	M	F
Austria	11	754	65	60	65	60	65	60	65	60	65	60	65	60
Germany	12	1499	65	65	65.1	65.1	65	65	65	65	65.5	65.5	65.7	65.7
Sweden	13	1117	65	65	65	65	65	65	65	65	65	65	65	65
Netherlands	14	624	65	65	65	65	65.2	65.2	65.5	65.5	65.8	65.8	66.3	66.3
Spain	15	1175	65	65	65	65	65	65	65	65	65	65	65	65
Italy	16	1022	65	60	66	62	66	62	66.6	65.6	67	66.6	62	62
France	17	1065	60	60	60.8	60.8	61.2	61.2	61.6	61.6	63.5	63.5	63.5	63.5
Denmark	18	1615	65	65	65	65	65	65	65	65	65	65	65.5	65.5
Greece	19	195	65	65	62	62	62	62	62	62	62	62	62	62
Switzerland	20	1122	65	64	65	64	65	64	65	64	65	64	65	64
Belgium	23	1715	65	65	65	65	65	65	65	65	65	65	65	65
Israel	25	409	67	62	67	62	67	62	67	62	67	62	67	62
Czech Republic*	28	1052	-	-	-	-	62.7	61.3	63	62.3	63.2	62.7	63.7	63.7
Poland	29	152	65	60	65	60	65.3	60.3	66	61	65	60.8	65	60
Luxembourg	31	306	60	60	60	60	60	60	62	62	62	62	62	62
Portugal	33	23	65	65	65	65	66	66	66.2	66.2	65.2	65.2	65.3	65.3
Slovenia	34	547	58	56	58	57.4	58.7	58.3	60	59.3	62	61.7	62	62
Estonia	35	1623	63	62	63	62	63	63	63	63	63.3	63.3	63.8	63.8
Croatia*	47	324	-	-	-	-	65	61	65	61.5	65	62	65	62.5
Total		16339												

*No information available regarding retirement age before 2014. Countries joined SHARE at later moment in time.

Table S3. Changes in memory before and after an early exit from paid employment due to disability: the role of job-effort reward imbalance among 281 ageing workers.

		Memory function	
		Subjective (1 – 100)	Objective (0 – 100)
	N (%)	β (SE)	β (SE)
**Effort-reward imbalance**	142 (50.5)		
Annual change before transition		-1.49 (0.81)	**-1.10 (0.53)**
Step-change during transition		-2.96 (3.10)	1.78 (1.80)
Annual change after transition		0.70 (0.59)	0.09 (0.36)
**No effort-reward imbalance**	139 (49.5)		
Annual change before transition		0.47 (0.79)	0.27 (0.45)
Step-change during transition		**-7.82 (3.40)**	-0.23 (1.70)
Annual change after transition		0.14 (0.70)	-0.74 (0.38)

Associations are adjusted for age, sex and education level.
Bold: Statistically significant at the 0.05 level.

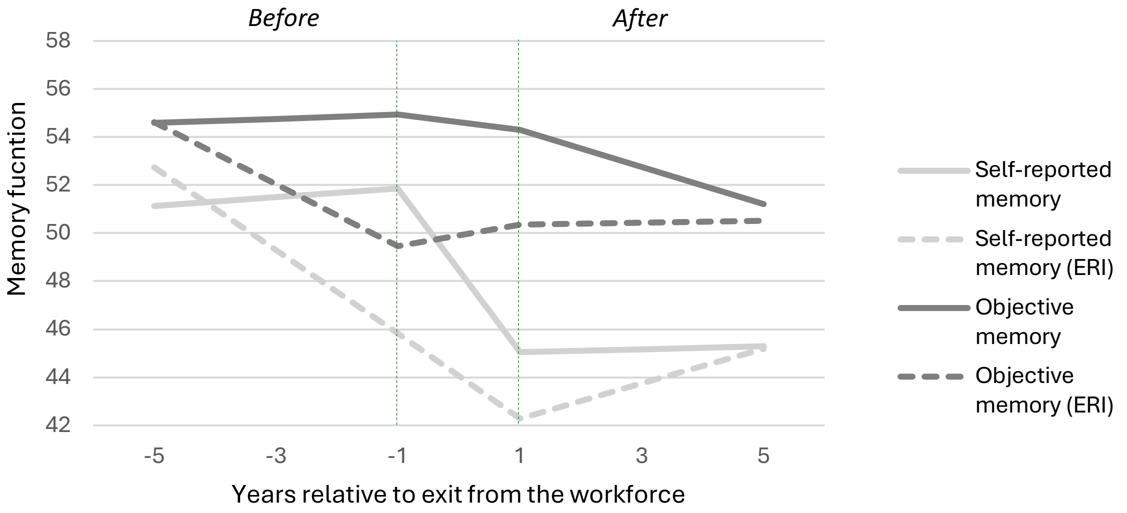

Figure S2. Memory function trajectories among ageing workers exiting the workforce early due to disability: the impact of job effort-reward imbalance (unadjusted)

**Table S4.** Changes in memory before and after early exit from paid employment due to unemployment: the role of job-effort reward imbalance among 552 ageing workers.			
		Memory function	
		Subjective (1 – 100)	Objective (0 – 100)
	N (%)	β (SE)	β (SE)
**Effort-reward imbalance**	269 (48.7)		
Annual change before transition		**-1.43 (0.64)**	-0.20 (0.37)
Step-change during transition		**-5.54 (2.35)**	1.29 (1.24)
Annual change after transition		0.72 (0.59)	-0.50 (0.28)
**No effort-reward imbalance**	283 (51.3)		
Annual change before transition		**-1.57 (0.59)**	0.46 (0.35)
Step-change during transition		1.03 (2.28)	1.22 (1.23)
Annual change after transition		-0.29 (0.48)	**-0.57 (0.28)**

Associations are adjusted for age, sex and education level.
Bold: Statistically significant at the 0.05 level.

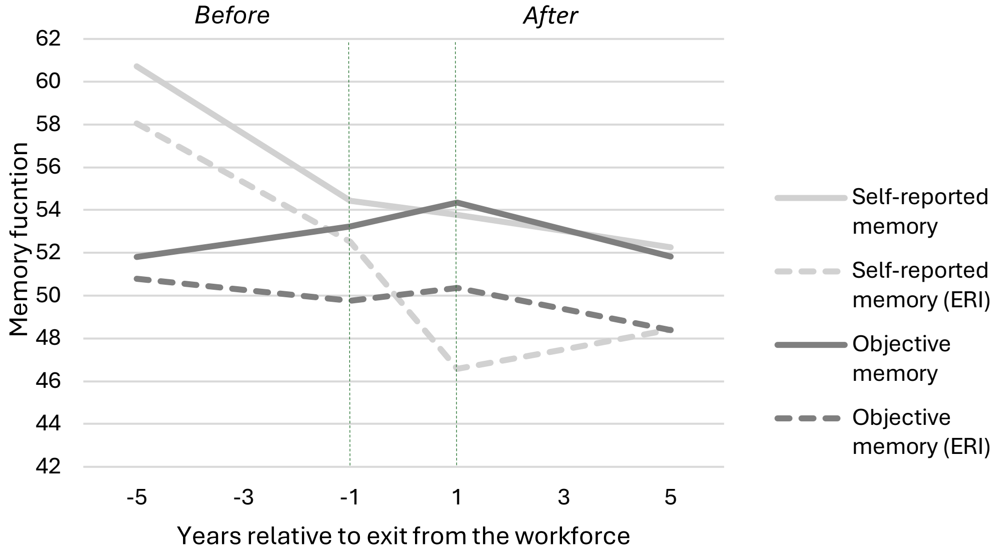

Figure S3. Memory function trajectories of ageing workers exit the workforce early due to unemployment: the impact of job effort-reward imbalance (unadjusted)
